# Sugar Sensing and Signaling in *Candida albicans* and *Candida glabrata*

**DOI:** 10.3389/fmicb.2019.00099

**Published:** 2019-01-30

**Authors:** Mieke Van Ende, Stefanie Wijnants, Patrick Van Dijck

**Affiliations:** ^1^Laboratory of Molecular Cell Biology, Institute of Botany and Microbiology, Department of Biology, KU Leuven, Leuven, Belgium; ^2^VIB-KU Leuven Center for Microbiology, Leuven, Belgium

**Keywords:** *Candida albicans*, Candida glabrata, Saccharomyces cerevisiae, sugar sensing, sugar transport, Snf3/Rgt2-Hgt4, Gpr1/Gpa2, Snf1/Mig1

## Abstract

*Candida* species, such as *Candida albicans* and *Candida glabrata*, cause infections at different host sites because they adapt their metabolism depending on the available nutrients. They are able to proliferate under both nutrient-rich and nutrient-poor conditions. This adaptation is what makes these fungi successful pathogens. For both species, sugars are very important nutrients and as the sugar level differs depending on the host niche, different sugar sensing systems must be present. *Saccharomyces cerevisiae* has been used as a model for the identification of these sugar sensing systems. One of the main carbon sources for yeast is glucose, for which three different pathways have been described. First, two transporter-like proteins, *Sc*Snf3 and *Sc*Rgt2, sense glucose levels resulting in the induction of different hexose transporter genes. This situation is comparable in *C. albicans* and *C. glabrata*, where sensing of glucose by *Ca*Hgt4 and *Cg*Snf3, respectively, also results in hexose transporter gene induction. The second glucose sensing mechanism in *S. cerevisiae* is via the G-protein coupled receptor *Sc*Gpr1, which causes the activation of the cAMP/PKA pathway, resulting in rapid adaptation to the presence of glucose. The main components of this glucose sensing system are also conserved in *C. albicans* and *C. glabrata*. However, it seems that the ligand(s) for *Ca*Gpr1 are not sugars but lactate and methionine. In *C. glabrata*, this pathway has not yet been investigated. Finally, the glucose repression pathway ensures repression of respiration and repression of the use of alternative carbon sources. This pathway is not well characterized in *Candida* species. It is important to note that, apart from glucose, other sugars and sugar-analogs, such as *N*-acetylglucosamine in the case of *C. albicans*, are also important carbon sources. In these fungal pathogens, sensing sugars is important for a number of virulence attributes, including adhesion, oxidative stress resistance, biofilm formation, morphogenesis, invasion, and antifungal drug tolerance. In this review, the sugar sensing and signaling mechanisms in these *Candida* species are compared to *S. cerevisiae.*

## Introduction

**Figure 1:**
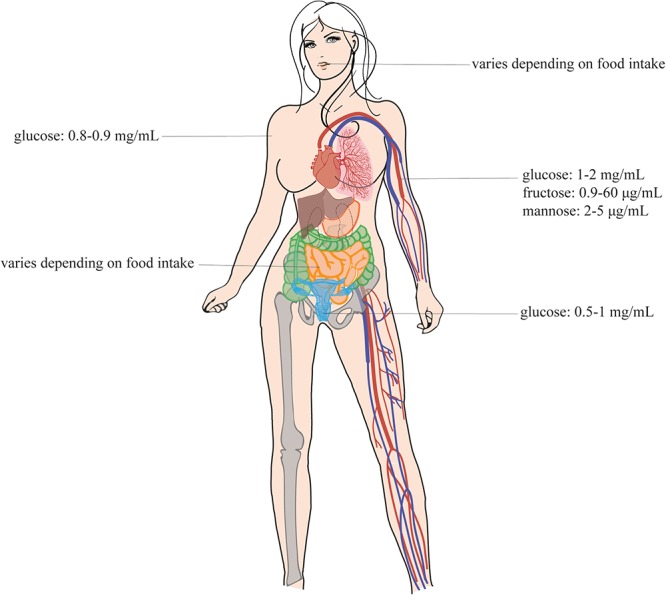
Sugar concentrations in the human body. This figure shows the sugar concentrations at the different infection sites of the Candida species. These pathogens are present at different sites of the human host, namely, in the mouth, in the gut, in the vagina, in the blood, and on the skin (Sone et al., 2003; Ehrstrom et al., 2006; Groenendaal et al., 2010; Laughlin, 2014). This figure is adapted from the review of Gulati and Nobile (2016). Permission was given to reuse this figure.

Over the last decades, the incidence of fungal infections has tremendously increased (Arendrup, 2013; Enoch et al., 2017). For every individual, there is a reasonable chance to suffer from a superficial fungal infection at least once during their lifetime. Due to organ transplants, catheter use and the redundant and prophylactic use of antibiotics, there is a higher number of at-risk people, which causes an increase in both superficial and systemic infection rate of these pathogenic fungi (Pfaller and Diekema, 2007; Arendrup, 2010; Yapar, 2014). From the pool of fungal infections, the most frequently isolated species are *Candida sp.* of which *Candida albicans* is the most prominent one (Guinea, 2014; Goemaere et al., 2018). Furthermore, the introduction of the antifungal fluconazole caused an increase in infections initiated by the inherently more fluconazole tolerant species, *Candida glabrata* (Pfaller et al., 2005; Diekema et al., 2012; Pfaller et al., 2014). *C. glabrata* is often found in the environment (Greppi et al., 2015; Morrison-Whittle et al., 2018), while *C. albicans* is only rarely found outside the mammalian host. Recently some studies showed its presence in chickens and turkeys and on old oaks (Bensasson et al., 2018; Liu et al., 2018; Sokół et al., 2018). The differences in lifestyle between *C. albicans* and *C. glabrata* are due to their different genomic evolution. *C. albicans* diverged from *S. cerevisiae* about 300 million years ago (Hedges et al., 2015). Therefore, these species are fairly different from each other with as most obvious example the different usage of the CUG codon (Santos and Tuite, 1995). It is estimated that about 100–150 million years ago, *S. cerevisiae* underwent a whole genome duplication (WGD) (Sugino and Innan, 2005). It is after this that *C. glabrata* has diverged from *S. cerevisiae* as this pathogen shows remnants of the WGD (Herrero, 2005). Therefore, *C. glabrata* and *S. cerevisiae* are phylogenetically much closer related to each other than to *C. albicans*. This difference in lifestyles also has an influence on their nutrient sensing mechanisms. *C. glabrata* is able to sense the broad range of nutrient availability which varies tremendously between host and environment. In the human host, the fungal cells proliferate under both nutrient-rich and nutrient-poor conditions; however, the range of nutrient concentrations is smaller (Figure 1). The adaptation to different environments is the key to successful survival of these pathogens (Brock, 2009; Fleck et al., 2011).

**Figure 2:**
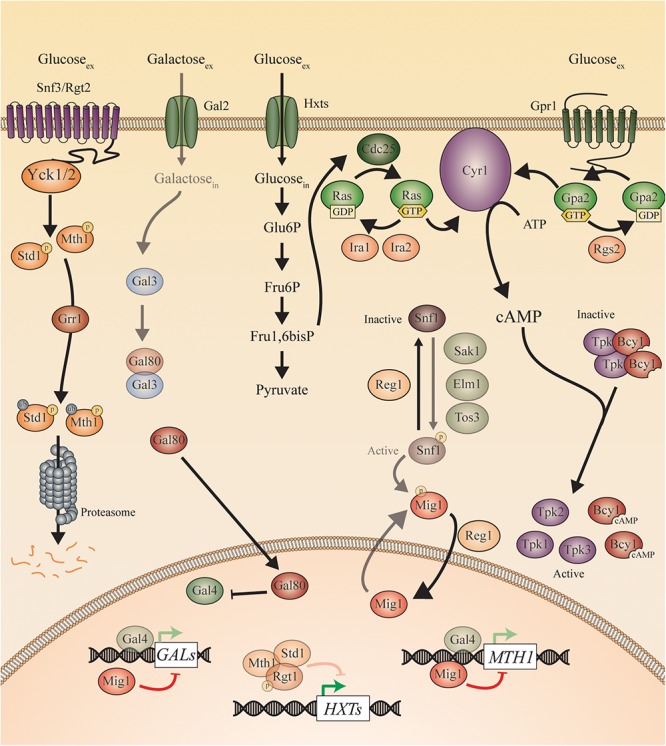
Sugar sensing and signaling in *S. cerevisiae*. The sugar-sensing network in *S. cerevisiae*. The faded arrows and proteins represent the situation when sugars are absent. All proteins are represented as circles. Activation is represented with green arrows, while red arrows indicate inhibition. When the ScSnf3/ScRgt2 receptors sense glucose, ScYck1 phosphorylates ScStd1 and ScMth1. These are subsequently ubiquitinated by ScGrr1 and broken down by the proteasome. In the absence of glucose, they function together with ScRgt1 to repress transcription of the ScHXT genes. The ScHxt transporters allow glucose to enter the cells after which this monosaccharide is converted to pyruvate during glycolysis. One of the intermediates of this pathway is fructose 1,6 bisphosphate which is found to activate ScCdc25. In this way, ScCdc25 allows the exchange of GDP to GTP on the ScRas proteins. ScRas is now activated and stimulates ScCyr1. Next to activation by ScRas, ScCyr1 needs the stimulus coming from ScGpr1 to be fully activated. ScGpr1 is activated by sensing extracellular glucose which allows the exchange of GDP to GTP on the ScGpa2 proteins. ScGpa2-GTP activates ScCyr1. ATP is converted into cAMP after complete activation of ScCyr1. cAMP binds to the regulatory subunits of PKA (Bcy1) thereby causing them to release the catalytic subunits (ScTpk1, 2, and 3) causing PKA activation. The absence of glucose triggers the phosphorylation of ScSnf1 by ScSak1, ScElm1, and ScTos3. The activated ScSnf1 protein phosphorylates ScMig1, preventing this protein to enter the nucleus. In the presence of glucose, both ScSnf1 and ScMig1 are dephosphorylated by ScReg1, allowing ScMig1 to enter the nucleus and function as a transcriptional repressor of the ScGAL genes and ScMTH1. When galactose is present (in the absence of glucose), it can enter the cells via the ScGal2 permease. Galactose can bind to ScGal3, which sequesters ScGal80 and prevents the latter to bind ScGal4. ScGal4 can thereby actively function in the nucleus as a transcription factor, inducing the expression of the ScGAL genes and of ScMTH1.

**Figure 3:**
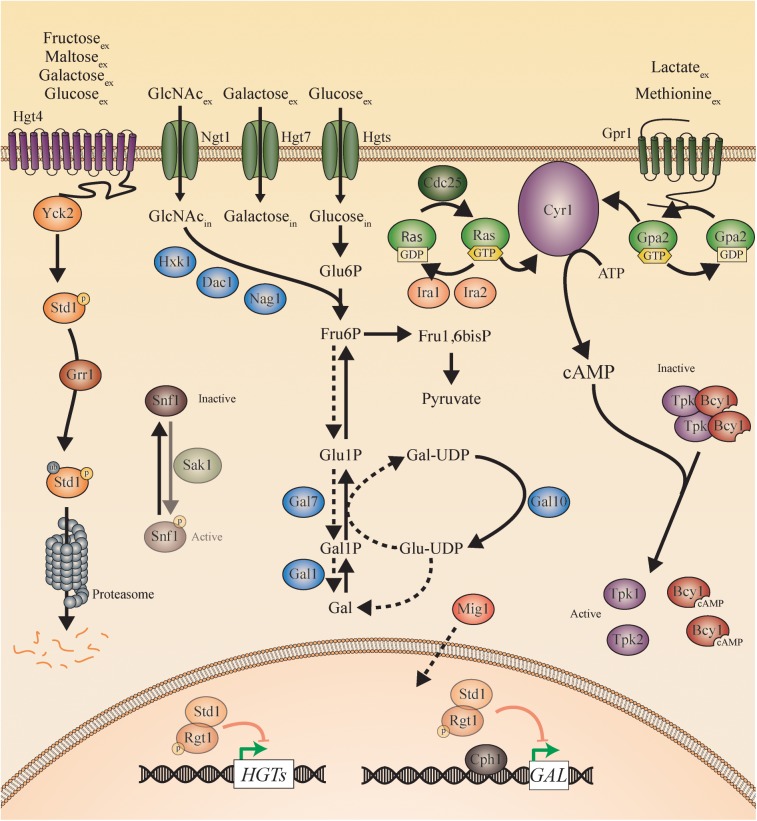
Sugar sensing and signaling in *C. albicans*. The sugar-sensing network in *C. albicans*. The faded arrows and proteins represent the situation when sugars are absent. All proteins are represented as circles. Activation is represented with green arrows, while red arrows indicate inhibition. The dashed lines represent the hypothesis. When the CaHgt4 receptor senses glucose, galactose, maltose, or fructose, CaYck2 phosphorylates CaStd1. This is subsequently ubiquitinated by CaGrr1 and broken down by the proteasome. In the absence of a carbon source, CaStd1 functions together with CaRgt1 to repress transcription of the CaHGT genes. The CaHgt transporters allow sugars to enter the cells. When glucose enters the cells, it is converted to pyruvate during glycolysis. In the presence of extracellular glucose, an active CaCdc25 allows the exchange of GDP to GTP on the CaRas proteins. CaRas is now activated and stimulates CaCyr1. Next to activation by CaRas, CaCyr1 needs the stimulus coming from CaGpr1 to be fully activated. CaGpr1 is activated by sensing extracellular molecules, presumably methionine or lactate, which allows the exchange of GDP to GTP on the CaGpa2 proteins. CaGpa2-GTP activates CaCyr1. ATP is converted into cAMP after complete activation of CaCyr1 by CaRas and by CaGpa2. cAMP binds to the regulatory subunits of PKA (CaBcy1) thereby causing them to release the catalytic subunits (CaTpk) causing PKA activation. When *N*-acetylglucosamine enters the cells via CaNgt1, it can be metabolized to fructose-6-phosphate by CaHxk1, CaDac1, and CaNag1. The hypothesis is that fructose-6-phosphate can be converted into galactose by the action of the CaGal enzymes. The galactose metabolism in *C. albicans* remains a mystery, but the expression of the CaGAL genes is found to be regulated by CaCph1 and CaRgt1. CaSnf1 can be phosphorylated by CaSak1 but no more in this pathway has been revealed.

To elucidate the nutrient-induced signal transduction pathways in human fungal pathogens, the model yeast species *S. cerevisiae* has been used extensively. Three different glucose-sensing pathways have been described in *S. cerevisiae*. First, glucose binds to either the high affinity sensor *Sc*Snf3 or the low affinity sensor *Sc*Rgt2, which results in the induction of hexose transporter (*HXT*) genes (Ozcan et al., 1996). Second, glucose also binds to the G-protein coupled receptor (GPCR) *Sc*Gpr1 resulting in the activation of its G-protein *Sc*Gpa2 and subsequent activation of adenylate cyclase (*Sc*Cyr1). This leads to an increase in cAMP levels and the activation of protein kinase A (PKA; Rolland et al., 2000; Thevelein et al., 2000). Full activation of this pathway also requires sugar phosphate-induced activation of *Sc*Cdc25 and *Sc*Ras (Toda et al., 1985; Rolland et al., 2000; Thevelein et al., 2000; Peeters et al., 2017). Once inside the cells, glucose causes repression of genes responsible for gluconeogenesis and respiration via the glucose repression pathway (Kayikci and Nielsen, 2015). This last one also has a negative effect on the expression of genes involved in the use of alternative carbon sources. For example, the *GAL* genes are repressed by the transcriptional inhibitor *Sc*Mig1, which is activated by high levels of glucose. This hinders the use of galactose as a carbon source when glucose is present in the medium. *Candida* species show many similarities with these pathways found in *S. cerevisiae* suggesting a same ancestor. However, also many differences in sensing and signaling have been described. This is to be expected, as *Candida* species have the ability to turn virulent whereas baker’s yeast generally does not. For example, glucose functions as a morphogen to influence yeast-to-hyphae transition in *C. albicans* (Maidan et al., 2005b). Additionally, glucose and other sugars have an influence on the ability of *Candida* cells to form a biofilm (Ng et al., 2016; Pemmaraju et al., 2016). This is a “city of microbes” where microorganisms live densely populated and surrounded by an excreted extracellular matrix as protection. Furthermore, sensing sugars is important for other virulence attributes, including adhesion, oxidative stress resistance, invasion, and antifungal drug tolerance (Vargas et al., 1993; Jin et al., 2004; Rodaki et al., 2009; Manns et al., 2014). In this review, we describe the different sugar sensing mechanisms in *C. albicans* and *C. glabrata* and compare them to the situation in *S. cerevisiae*. This is shown in Figure 2 for *S. cerevisiae* and in Figure 3, 4 for *C. albicans* and *C. glabrata*, respectively.

**Figure 4:**
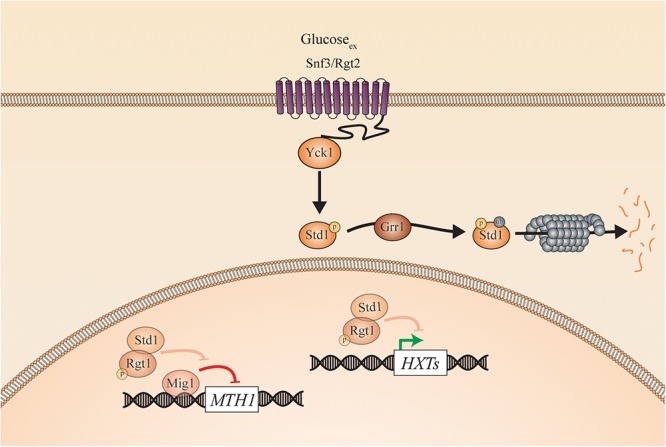
Sugar sensing and signaling in *C. glabrata*. The sugar-sensing network in *C. glabrata*. The faded arrows and proteins represent the situation when sugars are absent. All proteins are represented as circles. Activation is represented with green arrows, while red arrows indicate inhibition. When the CgSnf3/CgRgt2 receptors sense glucose CgYck1 phosphorylates CgStd1. This is subsequently ubiquitinated by CgGrr1 and broken down by the proteasome. In the absence of glucose, CgStd1 functions together with CgRgt1 to repress transcription of the CgHXT genes. The CgHxt transporters allow glucose to enter the cells.

## Glucose Sensing and Signaling

As glucose is the most abundant hexose sugar on earth, it is the preferred carbon source for many species. Due to its central role in metabolism, glucose functions both as a ligand for receptors and as a substrate for transporters (Towle, 2005). As a result, glucose serves as starting material for the biosynthesis of other biomolecules as well as for energy production via fermentation or respiration (Verduyn, 1991; Rodrigues et al., 2006).

### Sugar Receptor-Repressor (SRR) Pathway

*Saccharomyces cerevisiae* encodes 17 hexose transporters which all have different affinity and kinetic properties in response to glucose (Ozcan and Johnston, 1995). Furthermore, they all consist out of 12 transmembrane domains (TMDs; Ozcan and Johnston, 1999). Two of these proteins, *Sc*Snf3 and *Sc*Rgt2, are not able to transport glucose, but sense this sugar. Therefore, they have an additional long C-terminal extension compared to the sugar transporters (Ozcan and Johnston, 1999). *Sc*Snf3 is the high-affinity sensor while *Sc*Rgt2 responds to higher levels of glucose (Ozcan et al., 1996; Boles, 2003). In the presence of glucose, the transcription factors, *Sc*Std1 and *Sc*Mth1, are recruited to the cytoplasmic tail of *Sc*Snf3 or *Sc*Rgt2 where they are phosphorylated by the yeast casein kinase *Sc*Yck1 (Schmidt et al., 1999; Lafuente et al., 2000). The phosphorylated *Sc*Mth1 and *Sc*Std1 are subsequently ubiquitinated by *Sc*Grr1 leading to degradation by the proteasome (Moriya and Johnston, 2004). This degradation prevents *Sc*Rgt1-*Sc*Mth1/*Sc*Std1 complex formation and relieves the repression of the glucose transporter and metabolism genes. In the absence of glucose, *Sc*Mth1 and *Sc*Std1 interact with *Sc*Rgt1 to repress genes involved in the glucose metabolism (e.g., *HXT*s) (see Figure 2). Interestingly, the Snf3/Rgt2 glucose sensors and the Rgt1-Mth1/Std1 transcriptional repression complex seem to be fungal specific (Todd and Andrianopoulos, 1997; Ozcan et al., 1998).

Even though glucose is of major importance for *C. glabrata*, the sugar receptor-repressor (SRR) pathway has not yet been investigated in detail in this species (Ng et al., 2016). Only 11 Hxts are identified in *C. glabrata* compared to 17 in *S. cerevisiae* and 20 in *C. albicans.* It is, however, possible that clinical *C. glabrata* isolates express more hexose transporters than the CBS138 laboratory strain because they have evolved within the human host. This hypothesis is based on recent findings regarding the emergence of antifungal resistance genes and the number of adhesins present in *C. glabrata* clinical isolates (Vale-Silva et al., 2017; Salazar et al., 2018). The glucose sensor *Cg*Snf3 was first characterized by the group of Than as the most important and high affinity sensor of environmental glucose concentrations (Ng et al., 2015). *Cg*Snf3 and *Cg*Rgt2 are 59 and 60% similar to their orthologs in *S. cerevisiae*, respectively. These putative glucose sensors also consist out of 12 TMDs and have a long cytoplasmic tail. In comparison to the situation in *S. cerevisiae*, *Cg*Snf3 is important for growth on low physiological glucose levels (0.01–0.1%). The sensor is less important for growth on high glucose levels (1–2%) as it is probably not expressed under these conditions. Deletion of *CgSNF3* results in downregulation of four out of nine hexose transporter genes (Ng et al., 2015). Furthermore, the disruption of *CgSNF3* leads to the downregulation of *CgYCK1*, *CgYCK2*, and *CgSTD1*. This suggests that they work downstream of this high affinity glucose sensor. A *Cgsnf3*Δ mutant is also more susceptible to phagocytosis. This indicates that this receptor is important in host niches with low glucose levels, like inside macrophages (Ng et al., 2015). Whether this is due to the fact that some high affinity glucose transporters or other downstream genes are not expressed remains to be investigated. However, the Cg*snf3*Δ mutant has no defect in the formation of biofilms. This suggests that other sensors must be necessary to sense the nutrient flow in this environment or that the glucose level is still sufficient (Ng et al., 2015). *CgRGT2* is responsive to high surrounding glucose concentrations, indicating its low affinity, similar to *Sc*Rgt2 (see Figure 4). These findings suggest a conserved glucose signaling pathway between *S. cerevisiae* and *C. glabrata.* From the little knowledge there is, it is speculated that this pathway plays an important role in the virulence of this pathogenic fungus.

In *C. albicans*, the glucose transporter gene family was first studied by the group of Shen (Fan et al., 2002). The 12 TMDs of *S. cerevisiae* are conserved in *C. albicans*. Twenty high affinity glucose transporters are identified in the latter of which *CaHGT9*, *CaHXT10*, *CaHGT12*, and *CaHGT17* are strongly expressed upon low glucose levels (0.2%) and repressed at 5% glucose. The other *CaHGT* genes are expressed in YPD medium (2% glucose). These transporter-like proteins are divided into two groups by performing a bootstrap neighbor-joining analysis: *CaHXT* (*CaHGT6*, *CaHGT7*, *CaHGT8*, and *CaHGT11*) and *CaSNF3* (*CaHGT4* and *CaHGT12*) (Fan et al., 2002). Even though it seems to be clear that *Ca*Hgt4 is a sugar sensor, similar to Snf3 in *S. cerevisiae* and *C. glabrata*, it is not yet clear whether *Ca*Hgt12 is a sensor and/or transporter (Fan et al., 2002; Brown et al., 2006). *Ca*Hgt4 has 56% identical and 73% similar residues compared to its orthologs *Sc*Snf3/*Sc*Rgt2. Also, *Ca*Hgt12 shows high similarity to these latter ones (48% identical and 68% similar residues) but lacks the C-terminal extension which is a hallmark for the sensors (Helmerhorst et al., 2002). In order to define the protein responsible for sugar sensing in *C. albicans*, both *CaHGT4* and *CaHGT12* were disrupted. The *Cahgt12*Δ mutant shows no growth defect on sugar media containing glucose, fructose, or mannose. On the other hand, the *Cahgt4*Δ mutant shows a severe growth defect on fructose containing media and has problems growing on low concentrations of glucose and mannose (<0.2%) (Brown et al., 2006). The glucose concentrations which induce expression of *CaHGT4* are comparable with glucose concentrations present in human serum. Furthermore, the *Cahgt4*Δ mutant is also affected in its growth on glucose containing media in the presence of Antimycin A, a compound that inhibits respiration (Helmerhorst et al., 2002). These data suggest that *Ca*Hgt4 is a protein essential for growth on low levels of fermentable carbon sources and is important during hypoxic conditions (Brown et al., 2006). Upon sensing glucose, *Ca*Hgt4 signals downstream toward the transcription of several *CaHGT*s. In the *Cahgt4*Δ mutant, *CaHGT12*, *CaHGT7*, and *CaHXT10* are 10 times less expressed compared to their expression level in the *Ca*Hgt4 constitutively signaling strain (Brown et al., 2006). The promoters of these genes all contain six *Ca*Rgt1 binding sites causing constitutive repression when *CaHGT4* is deleted. The *Cargt1*Δ mutant shows a fivefold increase in expression of these hexose transporter genes (Sexton et al., 2007). In the *Cahgt4*Δ *Cargt1*Δ double mutant, the expression of *CaHGT12*, *CaHXT10* and *CaHGT7* is restored. This shows, similar to the situation in *S. cerevisiae*, that *Ca*Rgt1 is operating downstream of *Ca*Hgt4, although it has only limited sequence similarity (Brown et al., 2006). Most of the similar residues are found in the zinc-cluster DNA binding domain in the N-terminus of the protein (Sexton et al., 2007). As low levels of glucose are known to induce morphogenesis, it is expected that disrupting *CaHGT4* affects this virulence trait (Hudson et al., 2004). Indeed, the *Cahgt4*Δ mutant has a defect in filamentation, whereas the constitutively signaling form of *Ca*Hgt4 results in hyper filamentation (Brown et al., 2006). Furthermore, mice infected with the *Cahgt4*Δ mutant survive longer than mice infected with the wild type *C. albicans* strain. This shows the importance of this glucose sensor for virulence. As *Ca*Rgt1 signals downstream of *Ca*Hgt4, it is likely that repression of the hexose transporter genes by *Ca*Rgt1 also has an effect on filamentation (Maidan et al., 2005a). Indeed, the double *Cahgt4*Δ *Cargt1*Δ mutant shows hyper filamentation, similar to the phenotype observed in the strain containing the constitutively signaling form of Hgt4 (Brown et al., 2006; Sexton et al., 2007). Regarding the *Sc*Mth1 and *Sc*Std1 proteins, only one ortholog is present in *C. albicans*: *CaSTD1*, which is regulated in a similar way as in *S. cerevisiae* (phosphorylation-dependent proteolytic degradation). This suggests that *ScMTH1* occurred in *S. cerevisiae* by WGD after the split of the CUG clade and the *Saccharomyces* lineages (Brown et al., 2009).

Comparing the *Cgsnf3*Δ mutant with the *C. albicans Cahgt4*Δ mutant, the effect for growth on low glucose concentrations is different. The *Ca*Hgt4 sensor is important for fermentative growth while *Cg*Snf3 is essential under both respiratory and fermentative conditions (Brown et al., 2006; Ng et al., 2015). Therefore, the group of Than suggests that this high affinity sensor is more essential in *C. glabrata* than in *C. albicans.* This can be due to the fact that *C. glabrata* is Crabtree positive and prefers fermentation over respiration and produces ethanol in the presence of oxygen (Van Urk et al., 1990). As fermentation is the less favorable way to provide the cells with energy (fermentation: 2 ATP/glucose; respiration: 38 ATP/glucose), it is obvious that *C. glabrata* needs two specific glucose sensors to increase the glucose uptake. Thereby, it is able to differentiate the expression of sugar transporters and fulfill its ATP demand (Ng et al., 2015). Also, the differences in lifestyles between *C. glabrata* and *C. albicans* are a possible explanation for their different glucose sensors. *C. albicans* may have more co-evolved with the human host and as no high levels of glucose are present inside the human body, the low-affinity glucose sensor seems unnecessary. *C. glabrata* is more similar to *S. cerevisiae*, as these species are phylogenetically more closely related and they experience high and low glucose levels in nature. However, since both *C. glabrata* and *C. albicans* occur as human fungal pathogens, it is expected that more similarities in their sugar sensing mechanisms will be found. To elucidate this, further research on the SRR pathway in *C. glabrata* is necessary.

### Sugar Sensing and Signaling Toward PKA

A second mechanism by which *S. cerevisiae* is able to sense glucose and subsequently activate PKA is via the GPCR system, consisting of *Sc*Gpr1 and *Sc*Gpa2. The *Sc*Gpr1 receptor is able to sense both sucrose and glucose. Its affinity for glucose is very low, namely, a Ka of 75 mM (Rolland et al., 2000). This is related to the function of this GPCR in stimulating the switch between respirative/gluconeogenic growth and fermentative growth which only happens at glucose concentrations above 20 mM (Thevelein, 1991; Rolland et al., 2000). Other fermentable sugars like fructose, mannose, and galactose do not activate *Sc*Gpr1. However, mannose has an antagonistic effect on this sensor. Glucose, sucrose, and mannose all have very similar structures, so probably the position of the hydroxyl group on the second carbon atom is crucial for the activation of *Sc*Gpr1 (Lemaire et al., 2004). When *Sc*Gpr1 senses glucose or sucrose, it is able to activate *Sc*Gpa2. In its GTP bound state, *Sc*Gpa2 is able to activate *Sc*Cyr1, which is peripherally bound to the plasma membrane and converts ATP into cAMP (Uno et al., 1985; Nakafuku et al., 1988; Mitts et al., 1990; Kraakman et al., 1999). Increased cAMP levels subsequently activate PKA by binding to the regulatory subunit *Sc*Bcy1 releasing the catalytic subunits *Sc*Tpk1, 2, and 3. However, full activation of this pathway by glucose also requires glucose transport and its phosphorylation by one of the hexose kinases (Rolland et al., 2000; Thevelein et al., 2000). The molecular mechanism of this activation remained unclear until it was recently shown that the glycolytic intermediate, fructose-1,6-bisphosphate (FbP), is able to bind *Sc*Cdc25 thereby activating Ras (Peeters et al., 2017). The level of FbP is known to be controlled by multiple pathways indicating that this metabolite is important for the response of the cells to different levels of glucose (Hers, 1984). Activated GTP-bound *Sc*Ras binds to *Sc*Cyr1, leading to the activation of PKA (Toda et al., 1985; Jacquet, 1997). Both pathways upstream of *Sc*Cyr1 are required for full activation of PKA (Figure 2).

In the *C. glabrata* genome, orthologs of *Sc*Gpr1 and *Sc*Gpa2 are present, but so far, they are not characterized. Recently, a FRET system has been developed to measure PKA activity as well as cAMP levels in *C. glabrata* cells. Here, it is shown, that after addition of glucose to derepressed cells, both the intracellular cAMP level increases and PKA is activated. These findings suggest that, similar to the situation in *S. cerevisiae*, glucose stimulates the activation of PKA via cAMP (Demuyser et al., 2018).

The GPCR system of *Sc*Gpr1 and *Sc*Gpa2 is also present in *C. albicans*, where *Ca*Gpr1 has 43% sequence identity with its *S. cerevisiae* ortholog. Furthermore, the main structures like the seven transmembrane regions and the second half of the third cytosolic loop are conserved (Miwa et al., 2004). However, it seems that in this species the *Ca*Gpr1 receptor does not sense sugars as the receptor is dispensable for the glucose-induced increase in cAMP levels. *Ca*Gpr1 is clearly not required for the glucose-induced cAMP increase. This increase depends on the *Ca*Cdc25 and *Ca*Ras2 protein (Maidan et al., 2005a). However, the GPCR system seems to be upstream of the PKA pathway as addition of exogenous cAMP restores the hyphal defect of the *Cagpr1*Δ mutant on solid hyphae-inducing media. This morphogenesis defect is also restored by expression of a constitutively active *CaGPA2* or by overexpression of *CaTPK1*, a catalytic subunit of PKA, indicating that *Ca*Gpr1 and *Ca*Gpa2 are upstream of the PKA pathway (Figure 3). This is further supported by the fact that the *Cagpr1*Δ mutant and the *Cagpa2*Δ mutant show the same morphogenesis phenotype as the *Cagpr1*Δ *Cagpa2*Δ double mutant (Maidan et al., 2005a). This raises the question about which ligand is able to activate *Ca*Gpr1. The lab of Van Dijck showed that methionine might be the ligand of *Ca*Gpr1 as this receptor is required for the methionine-induced and PKA mediated morphogenesis (Maidan et al., 2005a; Schrevens et al., 2018). Furthermore, it was shown that *Ca*Gpr1 internalizes in the presence of methionine, indicating ligand-induced internalization (Maidan et al., 2005a). Finally, physiological concentrations of methionine provide the strongest filamentation phenotype (Maidan et al., 2005b). However, more recently, it was shown that lactate may be the ligand of *Ca*Gpr1 and causes the internalization of this receptor (Ballou et al., 2016). More detailed receptor–ligand interactions need to be performed to elucidate the ligands of this receptor in *C. albicans*. To conclude, the exact mechanism by which glucose, methionine, and lactate integrate at the level of the Ras-cAMP/PKA pathway and how glycolysis may link with the cAMP/PKA pathway in regulating virulence remains to be investigated. Nevertheless, it was previously shown that glycolysis is upregulated during infections and that it plays an important role during pathogenicity of the fungus (Barelle et al., 2006). Since PKA controls different virulence factors, it is possible that the effect of glycolysis on the virulence factors is signaled via the cAMP/PKA pathway.

### Glucose Repression Pathway

A third important pathway in glucose sensing is the glucose repression pathway. This pathway has a negative effect on the use of alternative carbon sources and respiration (Gancedo, 1998; Carlson, 1999; Hedbacker and Carlson, 2008). *S. cerevisiae* has a very efficient glucose repression system. When glucose is present, the utilization of alternative carbon sources is completely blocked (Gancedo, 1992). For example, the gene expression of enzymes working in the gluconeogenic pathway is repressed (Mercado et al., 1991; Mercado and Gancedo, 1992). In addition, also the degradation of mRNAs of those genes accelerates (Mercado et al., 1994; Yin et al., 1996). In this way, the only used carbon source is glucose. An important player in this pathway is *Sc*Snf1. This is a serine threonine protein kinase and the yeast ortholog of the mammalian AMPK protein. Upon glucose depletion, this protein is activated due to phosphorylation by three upstream kinases (*Sc*Sak1, *Sc*Tos3, and *Sc*Elm1) (Ludin et al., 1998). Phosphorylated *Sc*Snf1 has an indirect effect on the use of alternative carbon sources, by phosphorylating *Sc*Mig1, whereby the latter can no longer translocate to the nucleus. This causes the derepression of genes involved in the utilization of other sugars. In the presence of glucose, the dephosphorylation and subsequent inactivation of *Sc*Snf1 is stimulated by *Sc*Reg1 (Ludin et al., 1998). At the same time, *Sc*Mig1 can be dephosphorylated by *Sc*Reg1 and migrates from the cytoplasm to the nucleus causing repression of certain genes (Treitel and Carlson, 1995; Hardie et al., 1998; Cottier et al., 2017; Shashkova et al., 2017; Figure 2). *Sc*Mig1 has, for example, an inhibitory effect on *ScSUC2*, *ScGAL1*, *ScGAL4* and on the gluconeogenic genes (Nehlin and Ronne, 1990; Griggs and Johnston, 1991; DeVit and Johnston, 1999). *Sc*Snf1 and *Sc*Mig1 also have an effect on the expression of the high affinity glucose sensor *Sc*Snf3 and the high affinity transporters *Sc*Hxt2 and *Sc*Hxt4 (Ozcan and Johnston, 1999; Kaniak et al., 2004). There is no expression of these two transporters in a *Scsnf1*Δ mutant due to the constant repression by *Sc*Mig1 (Ozcan and Johnston, 1995). This indicates that *Sc*Snf1 is essential for the derepression of high affinity transport (Bisson, 1988).

Also *C. glabrata* has a *Sc*Snf1 ortholog with high similarity to its orthologs in *S. cerevisiae* and *C. albicans* (Celenza and Carlson, 1986; Petter et al., 1997). The deletion of *CgSNF1* causes the inability to use trehalose as a carbon source. A typical character trait for *C. glabrata* is that it solely uses glucose and trehalose as fermentable carbon sources. This finding indicates that also here, compared to the situation in *S. cerevisiae*, the use of alternative sugars is repressed in a *Cg*Snf1-dependent manner (Petter and Kwon-Chung, 1996).

In contrast to *S. cerevisiae*, *C. albicans* is able to use alternative carbon sources in the presence of glucose. This is due to posttranscriptional rewiring during the evolution of this pathogen (Sandai et al., 2012). Glucose still causes the degradation of certain gene transcripts involved in the metabolism of these other carbon sources, but the protein levels stay at a constant level. For example, isocitrate lyase (Icl1), a protein working in the glyoxylate cycle, is not ubiquitinated in the presence of glucose. On the other hand, when *ScICL1* is expressed in *C. albicans*, *Sc*Icl1 is degraded upon glucose addition. This indicates that the ubiquitin-dependent catabolite inactivation machinery is still present in *C. albicans*. However, enzymes working in the gluconeogenic and glyoxylate pathway of this pathogen are missing this ubiquitination site present in their orthologs in *S. cerevisiae.* Therefore, they are not degraded when glucose is present (Sandai et al., 2012). From further research, it became clear that the absence of this ubiquitination site is important for the virulence of *C. albicans*. When such a site is added to *Ca*Icl1, it is degraded in the presence of glucose and the cells have a reduced ability to colonize the gastrointestinal (GI) tract and to cause a systemic infection in mice. This indicates that the colonization and infection of the human host are enhanced by flexible carbon utilization (Childers et al., 2016). An important player in this glucose repression pathway is *Ca*Snf1. This protein is essential for viability of this pathogen, which is not the case for baker’s yeast and *C. glabrata* (Petter and Kwon-Chung, 1996; Petter et al., 1997). *Ca*Snf1 is highly upregulated during stress conditions and plays a role in drug resistance, indicating its possible importance for virulence (Orlova et al., 2008). *Ca*Snf1 is activated via phosphorylation by *Ca*Sak1, an enzyme essential for metabolic adaptation and *in vivo* fitness. This indicates that the other two upstream kinases may not be present or active in *C. albicans*. Indeed, there is no ortholog of *Sc*Tos3 and an inactivating mutation of *CaELM1* does not reduce *Ca*Snf1 phosphorylation. This indicates that *Ca*Sak1 is most likely the only dedicated *Ca*Snf1 activating kinase (Ramirez-Zavala et al., 2017; Figure 3). In contrast to the situation in *S. cerevisiae*, *Ca*Mig1 has no consensus sequence for phosphorylation by *Ca*Snf1. This suggests that the activity of *Ca*Mig1 may be differently controlled than *Sc*Mig1 or that it has a different role in *C. albicans*. This is strengthened by the fact that *Ca*Mig1 is not differentially expressed on different carbon sources (Zaragoza et al., 2000). However, *Ca*Mig1 does have a differential effect on the expression of other genes in the presence of different carbon sources. This is shown by disruption of *Ca*Mig1, leading to a higher sensitivity to weak organic acids when grown on YPD compared to maltose containing media. This is probably due to the lack of repression of several transporter genes (Cottier et al., 2017). Nevertheless, *CaMIG1* disruption is found to have no effect on the expression of the *GAL* regulon, which is later refuted by the group of Pavelka, who shows an increased *CaGAL1* expression in the *Camig1*Δ mutant (Zaragoza et al., 2000; Cottier et al., 2017). In *S. cerevisiae*, *Sc*Mig1 recruits the *Sc*Tup1-*Sc*Cyc8 complex to repress glucose-sensitive promoters (Tzamarias and Struhl, 1995). Furthermore, *Sc*Tup1 also functions independently from *Sc*Mig1 (Tzamarias and Struhl, 1995). This seems to be similar in *C. albicans*. Here, *Ca*Mig1 functions both dependently and independently from *Ca*Tup1 (Murad et al., 2001). This is strengthened by the fact that the *Camig1*Δ mutant shows a different phenotype than the *Catup1*Δ mutant. A disruption in *CaTUP1* causes constitutive filamentation whereas the *Camig1*Δ mutant shows normal morphogenesis (Tzamarias and Struhl, 1995; Zaragoza et al., 2000). *Ca*Mig1 probably also translocates to the nucleus as it has a KMPPK sequence, which is also present in the N-terminal region of *Sc*Hxk2. This region is involved in catabolite repression and targeting of the *Sc*Hxk2 protein to the nucleus (Herrero et al., 1998; Zaragoza et al., 2000).

As mentioned above, there are similarities as well as differences in the glucose sensing and signaling pathways between *S. cerevisiae* and *Candida* species. The SRR pathway seems to be very similar, while the glucose repression pathway seems to work differently. Also, for the activation of the PKA pathway, there are both similarities and differences observed. In *C. albicans*, it seems that other nutrients are also able to activate PKA using the same sensor. This is probably due to the fact that the *Candida* species are pathogenic fungi and need to use all the available carbon sources to become a successful pathogen. It is not a surprise to observe these differences between signal transduction pathways in *S. cerevisiae* and *C. albicans* as similar rewiring has been described for a number of pathways including for example the galactose metabolism circuitry and the regulation of stress response (Martchenko et al., 2007a; Brown et al., 2014).

## Galactose Sensing

Galactose metabolism is strongly regulated by the type of carbon source present in the environment. Most of the genes from this pathway are clustered in the *ScGAL* regulon, which is a genetic switch for the utilization of galactose in the absence of glucose (Lohr et al., 1995; Bhat and Murthy, 2001; Malakar and Venkatesh, 2014). *S. cerevisiae* encodes three regulatory *ScGAL* genes (*ScGAL4*, *ScGAL80*, *ScGAL3*) and five structural *ScGAL* genes (*ScGAL1*, *ScGAL2*, *ScGAL7*, *ScGAL10*, *ScMEL1*), all belonging to the Leloir pathway (Leloir, 1951; Holden et al., 2003). In the absence of galactose, the transcriptional activation domain of *Sc*Gal4 is bound by *Sc*Gal80, thereby preventing the induction of downstream genes. When galactose enters the cells via the *Sc*Gal2 permease, it results in the binding of *Sc*Gal3 with *Sc*Gal80 and thereby relieving the inhibitory activity of *Sc*Gal80 on *Sc*Gal4. This results in the induction of the galactose metabolism genes leading to proper galactose utilization (Laughon and Gesteland, 1982; West et al., 1984; Johnston, 1987; Lohr et al., 1995; Platt and Reece, 1998; Peng and Hopper, 2002). *Sc*Gal4 also induces expression of *ScMTH1*, encoding a co-repressor necessary for *Sc*Rgt1 function. In this way, galactose inhibits glucose assimilation by repressing the *ScHXT* genes (Ren et al., 2000). On the other hand, the *ScGAL* genes are also responsive to glucose repression via the *Sc*Mig1 repressor. However, as stated above, when glucose levels are low, *Sc*Mig1 is phosphorylated by *Sc*Snf1 and leaves the nucleus to be broken down by the proteasome, relieving its repression of the *ScGAL* genes (Nehlin et al., 1991; Flick and Johnston, 1992; Figure 2).

The regulation of galactose metabolism in *C. albicans* seems to be rewired compared to the one seen in *S. cerevisiae*. Whereas most of the galactose structural genes (*CaGAL1*, *CaGAL10*, *CaGAL7*, *CaGAL2*) are conserved between *C. albicans* and *S. cerevisiae*, this is not the case for the galactose regulatory genes. No *ScGAL3* or *ScGAL80* orthologs seem to be present in the *C. albicans* genome. A strong conservation is observed for *Ca*Gal4, which shares 86% sequence identity with *Sc*Gal4. However, the activation domain and the negatively charged region, necessary for interaction with Gal80, is missing in *C. albicans* (Martchenko et al., 2007a,b). In contrast to the situation in *S. cerevisiae*, the disruption of *CaGAL4* does not change the expression of the *CaGAL* genes. Surprisingly, the *Ca*Hgt4 sensor is required as a low affinity galactose sensor that begins to respond to galactose at 0.6% (Brown et al., 2006, 2009). Since both glucose and galactose are sensed by the same sensor, intracellular signaling must be strictly regulated. However, it is found for wild type *C. albicans* cells that 94% of the genes induced in response to galactose are also induced in response to low or high levels of glucose (Brown et al., 2009). This result may be explained by the fact that heterologous expression of *Ca*Hgt4 in *S. cerevisiae* results in galactose-induced expression of *ScHXT* genes. This indicates that *Ca*Hgt4 is able to couple to the downstream signal transduction pathway as the endogenous *Sc*Snf3/*Sc*Rgt2 receptors are unable to sense galactose (Brown et al., 2009). The addition of galactose to *C. albicans* wild type cells induces expression of several genes listed in Supplementary Table S1. Addition of 2% galactose to glycerol grown *C. albicans* cells results in expression of the Leloir enzyme encoding genes (*CaGAL1*, *CaGAL7*, *CaGAL10*), genes involved in glucose metabolism (*CaHGT7*, *CaHXK2*, *CaMTH1*), and many others (*CaQDR1*, *CaAOX2*, *CaCMK1*) (Martchenko et al., 2007a; Brown et al., 2009). Most of the galactose-induced genes contain a *Ca*Rgt1-consensus site (5′-CGGANNA-3′) and show lower expression in a *Cacph1*Δ mutant, suggesting a coordinated regulation by *Ca*Rgt1 and *Ca*Cph1 (ortholog of Ste12 in *S. cerevisiae*) (Martchenko et al., 2007a). The expression of *CaSTD1* is unaffected by galactose, but is by glucose. This implies that galactose sensing is different between *C. albicans* and *S. cerevisiae* while glucose sensing is similar in both species (*Ca*Hgt4-*Ca*Std1-*Ca*Rgt1 or *Sc*Snf3-*Sc*Std1/*Sc*Mth1-*Sc*Rgt1). Galactose sensing in *C. albicans* is possibly due to a broad unspecific hexose sensor (*Ca*Hgt4) and an absent Gal4 pathway (Brown et al., 2009).

The galactose-sensing signal transduction pathway in *C. albicans* was one of the first pathways where a clear rewiring was observed at the transcriptional level when compared to the situation in *S. cerevisiae*. The difference in regulation of the Leloir genes in both fungi may be due to the fact that galactose plays an important role in certain virulence processes of *C. albicans*, namely, adhesion and biofilm formation, which are less important in *S. cerevisiae* (Jin et al., 2004; Martchenko et al., 2007a). In contrast to baker’s yeast, *C. albicans* uses glucose and galactose at the same time, which may be of importance when colonizing the GI tract of the human host in early stages of life (Cox, 1986; Sabina and Brown, 2009). At this time, glucose and galactose are present in high amounts due to milk intake (Grenov et al., 2016). *C. glabrata* is unable to metabolize galactose and does not encode any gene of the *GAL* regulon (Hittinger et al., 2004).

## Sensing of Other Sugars

### *N*-Acetyl Glucosamine

The amino-sugar *N*-acetylglucosamine (GlcNAc), which is present in the human host in mucosal layers, is used as a carbon and nitrogen source by *C. albicans.* GlcNAc is an important signaling molecule and is also the monosaccharide of chitin. Therefore, GlcNAc forms part of the fungal cell wall. GlcNAc is transported into the cells by the *Ca*Ngt1 transporter and is metabolized by *Ca*Nag1, *Ca*Hxk1, and *Ca*Dac1 into fructose-6-phosphate, which is further metabolized to pyruvate (Simonetti et al., 1974; Cardinali et al., 1997; Kumar et al., 2000; Leberer et al., 2001; Yamada-Okabe et al., 2001; Alvarez and Konopka, 2007). It is intriguing that next to galactose, GlcNAc also induces the expression of *CaGAL1*, *CaGAL7*, and *CaGAL10* (Kamthan et al., 2013). A disruption of *CaGAL10* shows a severely reduced expression of *CaGAL1* and *CaGAL7* in the presence of GlcNAc. This finding suggests an important role for *CaGAL10* in the GlcNAc mediated upregulation of the Leloir pathway genes. The induction of these genes peaks 90 min after the addition of GlcNAc whereas this expression maximum is already visualized 15 min after galactose addition (Kamthan et al., 2013). These data suggest that GlcNAc has an indirect effect on the induction of the *CaGAL* genes. Interestingly, intracellular galactose levels increase in cells growing in GlcNAc containing media which implies that GlcNAc induces the synthesis of the internal galactose, a trait already reported in *Escherichia coli* and *Kluyveromyces lactis* (Wu and Kalckar, 1966; Cardinali et al., 1997). This may then also explain the delayed and possibly indirect induction of the *CaGAL* genes. Even though *Ca*Cph1 is responsible for galactose induced expression, the transcriptional effect of GlcNAc remains unchanged in a *Cacph1*Δ mutant (Sexton et al., 2007; Kamthan et al., 2013; Figure 3).

Apart from the genes described above, many other genes are induced in the presence of GlcNAc (Supplementary Table S1) (Gunasekera et al., 2010). Several of them are involved in virulence (Shepherd et al., 1980; Singh et al., 2001; Huang et al., 2010; Noble et al., 2010). For example, GlcNAc seems to activate the cAMP/PKA pathway as this pathway is required for the GlcNAc-induced morphogenesis. *C. albicans* grows on GlcNAc as a sole carbon source which needs to be internalized to cause filamentation (Singh and Datta, 1979; Kumar et al., 2000; Alvarez and Konopka, 2007). On the other hand, the catabolism of GlcNAc is not required to induce morphogenesis, as the *Cahxk1*Δ *Canag1*Δ *Cadag1*Δ triple mutant is efficiently stimulated by GlcNAc to form hyphae (Naseem et al., 2011). Interestingly, GlcNAc induces a phenomenon called sugar-induced cell death (SICD; Du et al., 2015). This phenomenon was first identified in *S. cerevisiae* where the induction of glucose in water-cultured cells “tricks” the cells to enter an active cell cycle and leave the protective stationary phase. However, since there is a lack of other nutrients, they rapidly lose viability and undergo programmed cell death (apoptosis) (Granot and Snyder, 1991). *C. albicans* also undergoes apoptosis when it encounters different environmental stresses. The fact that GlcNAc can also induce SICD shows that this amino-sugar regulates multiple cellular programs in a coordinated manner and thereby maximizing the efficiency of nutrient use (Du et al., 2015). *S. cerevisiae* strains do not grow on GlcNAc as sole carbon source, but introducing the *C. albicans* GlcNAc catabolic genes allows baker’s yeast to use GlcNAc as a sole carbon source. This strain is useful in the research toward a renewable way of biofuel production (Wendland et al., 2009). This GlcNAc metabolism is not present in *C. glabrata*.

### Fructose and Mannose

Together with glucose, mannose and fructose belong to the top three of most preferred sugars for yeast species. In *S. cerevisiae*, fructose and mannose have similar sensing and transport mechanisms as glucose via the SRR pathway. Both sugars are sensed by *Sc*Snf3/*Sc*Rgt2 and the extracellular fructose/mannose level has an effect on the expression of the hexose transporter genes (Dietvorst et al., 2010). All hexose transporter genes which are induced by the *Sc*Snf3/*Sc*Rgt2 signaling pathway are able to transport fructose and mannose (Boles and Hollenberg, 1997). Seven transporters (*Sc*Hxt1–*Sc*Hxt7) are essential for growth on fructose/mannose at any concentration tested (Reifenberger et al., 1995; Liang and Gaber, 1996; Reifenberger et al., 1997).

This broad substrate sensing is conserved for the *C. albicans Ca*Hgt4 sensor. Apart from glucose, it also senses fructose and mannose as a *Cahgt4*Δ mutant is strongly impaired for growth on either sugar, with a more severe effect on fructose. The sensing of fructose and mannose induces the expression of seven of the 20 hexose transporters genes under which, *CaHGT12* and *CaHGT*7 (Brown et al., 2006; Luo et al., 2007; Brown et al., 2009). This last one is activated by low levels (0.04%) of fructose and mannose (Brown et al., 2009). It suggests that these transporters are more critical for fructose transport in comparison to their relevance for glucose and mannose transport (Brown et al., 2006). As a final remark, *C. glabrata* is unable to use fructose or mannose as a carbon source.

## Sugar and Virulence

Taken together, both similarities and differences are found in the sugar sensing and signaling mechanisms of *S. cerevisiae* and the *Candida* species. The most important observation is that *C. albicans* is able to use multiple carbon sources at the same time, while glucose represses the use of all other carbon sources in *S. cerevisiae* (Gancedo, 1992; Sandai et al., 2012). Probably, this is due to the metabolic flexibility of this pathogen, which is necessary to colonize the human body. The response of the cells to the nutrients present in the microenvironment is essential to become a successful pathogen (Rodaki et al., 2009).

Glucose has an effect on different virulence traits of both *C. albicans* and *C. glabrata*. One of the most important virulence factors of these pathogens is their ability to form biofilms. In this heterogenic community, the cells show a differentially active metabolism. Those which are in contact with glucose have an upregulation of glycolysis while others have an upregulated gluconeogenesis (Rodaki et al., 2009). During biofilm maturation, *C. albicans* forms hyphae, which are important for structural support, adherence to other cells and fulfillment of their nutrient needs (Chandra et al., 2001; Xu et al., 2014). Upon high levels of glucose, morphogenesis is repressed whereas hyphae formation is strongly induced from sugar levels lower than 0.25% (Maidan et al., 2005b). This indicates that sugar levels in biofilms are presumably below 0.25%. Depending on the environmental conditions, *Candida* cells disperse from the biofilm. During rich sugar conditions, there is more dispersion observed than under low sugar availability (Uppuluri et al., 2010). This suggests that biofilms are a survival strategy during poor nutrient conditions. Especially the carbon source present in the surroundings has a major effect on this process as the presence of glucose induces more dispersion than the presence of galactose (Uppuluri et al., 2010).

Second, another important virulence factor of *Candida* species is their high stress tolerance. Increasing glucose concentrations (0.01–1%) lead to upregulation of stress resistance genes in *C. albicans*, causing an increased resistance to azole antifungal drugs and osmotic stress (Rodaki et al., 2009; Ene et al., 2012). This is remarkable as *S. cerevisiae* downregulates its stress response genes in the presence of increasing concentrations of glucose (Rodaki et al., 2009). More comparable to the situation in *S. cerevisiae*, *C. glabrata* has a lower resistance against antifungal drugs upon increasing glucose levels. After treatment with amphotericin B, there was 50% less survival of the *C. glabrata* cells in the presence of 1% glucose then when less glucose (0.2%) was present (Ng et al., 2016). This indicates that this pathogen is more resistant due to a slower growth rate in the presence of low glucose levels (Ng et al., 2016). Additionally, *C. albicans* and *C. glabrata* are less susceptible for oxidative stress in the presence of increasing concentrations of glucose. *C. glabrata* is even more resistant than *C. albicans* as it tolerates 0.05 M of hydrogen peroxide, while this concentration damages the latter in the presence of 1% glucose (Rodaki et al., 2009; Ng et al., 2016). The mechanisms which link glucose to oxidative and osmotic stress resistance may be of importance during the infection process of *C. albicans* and *C. glabrata*. When entering the bloodstream, the cells are suddenly exposed to glucose, possibly leading to a fast induction of oxidative and osmotic stress resistance genes. This may be important for survival as, in the bloodstream, *Candida* cells are attacked by neutrophils, which try to kill off the pathogens by oxidative burst and low nutrient availability (Rodaki et al., 2009).

The glucose sensing pathways of *C. albicans* and *C. glabrata* described above are of importance for systemic infections. Many involved mutants have been tested in systemic mice models showing various results. It is known that the *Cahgt4*Δ mutant is not able to form hyphae. Consequently, these cells have a delayed virulence in mice. Where wild type cells can kill 90% of the mice after 5 days, the *Cahgt4*Δ mutants were only able to do this after 14 days (Brown et al., 2006). This indicates that the lack of this sugar sensor is not sufficient to render the cells completely avirulent. The *Cahgt4Δ Cargt1*Δ mutant shows a hyper-filamentous phenotype (Brown et al., 2006). This block in morphogenesis indicates that the double mutant will be impaired in virulence during a systemic infection. This has not yet been tested. Different components of the sugar signaling pathway toward PKA have been tested for their effect during systemic infections. Both *Ca*Gpr1 and *Ca*Gpa2 do not have any effect on the host survival. No difference in the mice survival rate was detected when mice were infected with the *Cagpa2*Δ mutant and the wild type. Mice infected with the *Cagpr1*Δ mutant showed a slight increase in survival of 20% compared to the wild type (Miwa et al., 2004; Maidan et al., 2005a). Moreover, it seems that *Ca*Cyr1 is very important during systemic infections since all mice infected with the *Cacyr1*Δ mutant could survive (Rocha et al., 2001). Finally, until now no components of the glucose repression pathway were tested for an effect on systemic infections.

From the findings presented above, it is clear that blood sugar levels have an effect on virulence by *Candida albicans* cells (Lorenz et al., 2004; Rodaki et al., 2009). Furthermore, *C. glabrata* cells isolated from the blood have a higher tendency toward biofilm formation than other cells (Ng et al., 2016). Also sugar related diseases have an effect on the virulence of these pathogens. For example, patients with diabetes have a higher chance of getting a systemic *Candida* infection compared to non-diabetic people (Rodaki et al., 2009). Furthermore, also a higher amount of oral *Candida* infections occur in people suffering from diabetes mellitus compared to healthy individuals (Zomorodian et al., 2016). This indicates that colonization and invasion by *Candida* species is enhanced via dietary glucose (Vargas et al., 1993). Constituently, we can conclude that the different sugar sensing and signaling pathways described above are important factors for both virulence and pathogenesis of *C. albicans* and *C. glabrata*.

## Concluding Remarks

As *Candida* species are an increasing problem, it is important to investigate their main virulence factors in order to find proper treatments. We focused on *C. albicans* and *C. glabrata*, because they are the two most isolated *Candida* species. However, in the last 10 years, there has been a big increase in other *Candida* species like *Candida auris* and *Candida krusei* (Jeffery-Smith et al., 2018). The problem with these species is that they show high antifungal resistance toward the current antifungal drugs which leads to major concerns. Further, *C. glabrata* is found to be inherently more resistant to azole drugs (Whaley and Rogers, 2016). Finally, more and more *C. albicans* clinical isolates show antifungal drug resistance (Whaley et al., 2016). This indicates the need for new antifungal drug targets and corresponding drugs. In this review, we show that sugar sensing and signaling are important for *Candida* species to turn virulent. Therefore, better understanding of the sugar sensing and metabolism pathways offer valuable antifungal drug targets. As stated, many of the players in these pathways are conserved between *S. cerevisiae*, *C. albicans*, and *C. glabrata*. Targeting these players result in a broad-spectrum antifungal drug against fungi. This kind of drug is desired by clinics and pharmaceutical companies, as nowadays one of the biggest issues with fungal infections is the diagnosis of the species (Perfect, 2017). For example, targeting fungal Gpr1 proteins will not lead to a fungistatic or fungicidal drug. However, this protein is important for morphogenesis and blocking this protein prevents the cells to turn virulent. This may be a better strategy for commensal fungi which are also present in a healthy human body, as it does not block normal cell growth (Brown et al., 2018). Other proteins may be less desired as many of them are highly conserved in human cells and thereby the corresponding drugs may have toxic effects.

In the future, these pathways should be unraveled in different pathogenic fungi and similarities should be marked. Similar players in different fungal species which are not conserved in humans are the desired drug targets nowadays. Furthermore, connections between the different pathways should be elucidated to better understand sugar signaling in pathogenic fungi and to find appropriate drug targets.

## Author Contributions

All authors contributed to the writing of the manuscript.

## Conflict of Interest Statement

The authors declare that the research was conducted in the absence of any commercial or financial relationships that could be construed as a potential conflict of interest. The handling Editor declared a past co-authorship with one of the authors PV.
