# Supplementary material for: Sugar Sensing and Signaling in Candida albicans and Candida glabrata
Source: Front Microbiol. 2019 Jan 30;10:99. doi: 10.3389/fmicb.2019.00099 (PMC6363656; doi:10.3389/fmicb.2019.00099)
Supplement: Supplementary file 1 [file Table_1.DOCX]

|  |  |  | **GALACTOSE** | **GLUCOSE** | **GlcNAc** |  |  |
| --- | --- | --- | --- | --- | --- | --- | --- |
| **NAME** | **ORF ID** | **ORTHOLOG IN *S. CEREVISIAE*** | **EXPRESSION** | **EXPRESSION** | **EXPRESSION** | **ANNOTATION** | **REFERENCE** |
| *HGT12* | orf19.7094 | *SNF3* | 6,74 ; 5,10 | Repressed |  | Glucose, Frutose, mannose transporter | Brown, 2009; Martchenko, 2007 |
| *GAL1* | orf19.3670 | *GAL1* | 6,1 ; 4,18 | Repressed | 2,756597523 | Galactokinase | Brown, 2009; Martchenko, 2007 |
| *GAL10* | orf19.3672 | *GAL10* | 6 ; 5,52 | Repressed | 4,611768522 | UDP-glucose-4epimerase | Brown, 2009; Martchenko, 2007; Gunasekera, 2010 |
| *GAL7* | orf19.3675 | *GAL7* | 5,9 ; 4,13 | Repressed | 2,617872676 | UDP-hexose-1-P uridyltransferase | Brown, 2009; Martchenko, 2007; Gunasekera, 2010 |
| *HXT5* | orf19.4384 | *ITR1* | 2,6 ; 3,76 | Repressed |  | Sugar transporter | Brown, 2009; Martchenko, 2007; Gunasekera, 2010 |
| *HGT2* | orf19.3668 | *RGT2* | 2 ; 7,75 | Repressed |  | Putative glucose transporter | Brown, 2009; Martchenko, 2007 |
| *HGT7* | orf19.2023 | *GAL2* | 32,2 | 30,8 |  | Putative glucose transporter | Brown, 2009 |
| *QDR1* | orf19.508 | *QDR1* | 18,6 | 56,1 |  | Antibiotic resistance transporter | Brown, 2009 |
| *AOX2* | orf19.4773 | */* | 13,7 | 8 |  | Alternative oxidase | Brown, 2009 |
| *FDH4.3* | orf19.1774 | *FDH1* | 13 | 18,1 |  | Predicted dehydrogenase | Brown, 2009 |
| *CRZ2* | orf19.2356 | *CRZ1* | 10,1 | 12,8 |  | Putative transcription factor | Brown, 2009 |
| *FET99* | orf19.4212 | *FET3* | 10 | 19,1 |  | Multicopper oxidase family | Brown, 2009 |
| *RHR2* | orf19.5437 | *GPP1* | 9,2 | 25,1 |  | Putative glycerol 3-phosphatase | Brown, 2009 |
| *RNR22* | orf19.1868 | *RNR2* | 8,1 | 25,4 |  | Ribonucleoside di-Phosphate reductase | Brown, 2009 |
| *FDH98* | orf19.1117 | *FDH1* | 7,8 | 6,4 |  | Protein similar to Candida boidinii formate dehydrogenase | Brown, 2009 |
| *TPO3* | orf19.4737 | *TPO2* | 7,4 | 16,4 | 1,525300351 | Possible polyamine tarnsporter | Brown, 2009; Gunasekera, 2010 |
| *C5_03510C_A* | orf19.6660 | *YHR131C* | 7,2 | 20,1 |  | Protein of unknown function | Brown, 2009 |
| *PDC11* | orf19.2877 | *PDC1* | 6,2 | 9,1 |  | Similar to pyruvate decarboxylase | Brown, 2009 |
| *ATO1* | orf19.6169 | *ATO2* | 5,62 |  |  | conserved hypothetical gene, DNA metabolism | Martchenko, 2007 |
| *HGT6* | orf19.2020 | *HXT6* | 5,3 | 3,2 |  | Putative glucose transporter | Brown, 2009 |
| *MNN22* | orf19.3803 | *MNN2* | 5,3 | 9,1 |  | Golgi Alpha-1,2-mannosyltransferase | Brown, 2009 |
| *FMA1* | orf19.6837 | *YIR035C* | 5,2 | 9,9 | 1,633092131 | Membrane associated protein | Brown, 2009; Gunasekera, 2010 |
| *HAK1* | orf19.6249 | */* | 5,1 | 3,4 |  | Putative potassium transporter | Brown, 2009 |
| *HXK2* | orf19.542 | *HXK2* | 5 | 8 |  | Hexokinase II | Brown, 2009 |
| *AQY1* | orf19.2849 | *AQY1* | 4,78 |  |  | Aquaporin | Martchenko, 2007 |
| *TYE7* | orf19.4941 | *TYE7* | 4,4 | 5,7 |  | Putative bHLH transcription factor | Brown, 2009 |
| *GDH3* | orf19.4716 | *GDH3* | 4,3 | 20,2 |  | NADP-glutamate dehydrogenase | Brown, 2009 |
| *CMK1* | orf19.5911 | *TDA1* | 4,1 | 5 |  | Ca2+/Calmodulin-dependent kinase | Brown, 2009 |
| *C1_11080W_A* | orf19.2308 | *PFK27* | 3,9 | 10,3 |  | Putative 6-phosphofructo-2-kinase | Brown, 2009 |
| *FET34* | orf19.4215 | *FET3* | 3,8 | 6,8 |  | Similar to multicopper ferroxidase | Brown, 2009 |
| *STP4* | orf19.909 | *STP3* | 3,8 | 5,7 |  | Putative transcription factor | Brown, 2009 |
| *AOX1* | orf19.4774 | */* | 3,7 | 3,3 |  | Alternative oxidase | Brown, 2009 |
| *ENT4* | orf19.6770 | *ENT4* | 3,6 | 4,4 |  | protein with ENTH Epsin domain | Brown, 2009 |
| *EHT1* | orf19.3040 | *EHT1* | 3,4 | 8,4 |  | Similar to Eht1p | Brown, 2009 |
| *CR_09140C_A* | orf19.7310 | *MSC1* | 3,33 |  |  | meiotic sister-chormatid recombination | Martchenko, 2007 |
| *HGT1* | orf19.4527 | *HXT11* | 3,32 |  | 1,872422077 | hexose transporter | Martchenko, 2007; Gunasekera, 2010 |
| *CAT2* | orf19.4591 | *CAT2* | 3,28 |  |  | carnitine acetyltransferase amino acid metabolism | Martchenko, 2007 |
| *C2_00760C_A* | orf19.2048 | */* | 3,2 | 3,4 |  | Proten of unknown function | Brown, 2009 |
| *C4_01800W_A* | orf19.4612 | *AIM2* | 3,2 | 14,5 |  | Protein with a dienelactone hydrolase domain | Brown, 2009 |
| *CRP1* | orf19.4784 | *CCC2* | 3,2 | 6,9 |  | Copper transporter | Brown, 2009 |
| *PFK1* | orf19.3967 | *PFK1* | 3,2 | 7 |  | Alpha-subunit of phosphofructokinase | Brown, 2009 |
| *PGA37* | orf19.3923 | */* | 3,2 | 1,1 |  | Putative GPI-anchored protein | Brown, 2009 |
| *ARP8* | orf19.3359 | *ARP8* | 3,13 |  |  | actin-related protein | Martchenko, 2007 |
| *PFK2* | orf19.6540 | *PFK2* | 3,1 | 5,7 |  | Beta-subunit of phosphofructokinase | Brown, 2009 |
| *JEN2* | orf19.5307 | *JEN1* | 3,05 |  |  | carboxylic acid transporter | Martchenko, 2007 |
| *GLK1* | orf19.734 | *GLK1* | 3,02 |  |  | aldohexose specific glucokinase | Martchenko, 2007 |
| *PHO15* | orf19.4444 | *PHO13* | 3 | 5,8 |  | 4-nitrophenyl phosphatase | Brown, 2009 |
| *AHP1* | [orf19.2762](http://www.candidagenome.org/cgi-bin/locus.pl?locus=orf19.2762&seq_source=C.%20albicans%20SC5314%20Assembly%2021) | *AHP1* | 2,9 | 6,3 |  | Putative alkyl hydroperoxide reductase | Brown, 2009 |
| *HSP30* | orf19.4526 | *HSP30* | 2,9 | 0,48 |  | Similar to heat shock protein | Brown, 2009 |
| *MIG1* | orf19.4318 | *MIG1* | 2,9 | 3,2 |  | Transcriptional repressor | Brown, 2009 |
| *UBC15* | orf19.5337 | *UBC13* | 2,9 | 3 |  | Ub-conjugation, DNA repair | Brown, 2009 |
| *ZSP12* | orf19.733 | *YBR287W* | 2,9 | 3,3 |  | Has domain(s) with predicted role in transmembrane transport and integral component of membrane localization | Brown, 2009 |
| *ALS4* | orf19.4555 | *SAG1* | 2,87 |  |  | agglutinin-like protein 4 | Martchenko, 2007 |
| *C2_00990W_A* | orf19.2024 | */* | 2,8 | 1,7 |  | Ortholog of Candida albicans WO-1 | Brown, 2009 |
| *ARG1* | orf19.7469 | *ARG1* | 2,7 | 4 |  | Similar to argininosuccinate synthase | Brown, 2009 |
| *GLK3* | orf19.1408 | */* | 2,7 |  |  | glucokinase | Martchenko, 2007 |
| *ZSP11* | orf19.6117 | *YBR287W* | 2,7 | 2,8 |  | predicted auxin family transmembrane transporter | Brown, 2009 |
| *HSP70* | orf19.4980 | *SSA4* | 2,66 |  |  | heat-shock protein 70 | Martchenko, 2007 |
| *XUT1* | orf19.2882 | */* | 2,63 |  |  | putative purine permease | Martchenko, 2007 |
| *C1_01140C_A* | orf19.3302 | *GAC1* | 2,6 | 2,8 |  | Putative type-1 protein phosphatase targeting subunit | Brown, 2009 |
| *C1_03750W_A* | orf19.1034 | *DAP1* | 2,6 | 8,1 |  | predicted cytochrome b5-like Heme/Steroid binding domain | Brown, 2009 |
| *C6_04420W_A* | orf19.2125 | */* | 2,6 | 3,9 | 1,464394326 | Protein of unknown function | Brown, 2009; Gunasekera, 2010 |
| *PHO113* | orf19.2619 | *PHO11* | 2,6 | 3,6 |  | Constitutive acid phosphatase | Brown, 2009 |
| *ATO2* | orf19.739 | *ATO2* | 2,58 |  |  | transporter involved in nitrogen utilization | Martchenko, 2007 |
| *C7_02230W_A* | orf19.6491 | */* | 2,57 |  |  | hypothetical protein | Martchenko, 2007 |
| *GLK1* | orf19.13 | */* | 2,51 |  |  | glucokinase, aldohexose specific | Martchenko, 2007 |
| *C6_03620C_A* | orf19.5730 | *FDC1* | 2,5 | 2,2 |  | Putative phenylacrylic acid decarboxylase | Brown, 2009 |
| *GPX2* | orf19.85 | *HYR1* | 2,5 | 2,25 |  | Similar to glutathione peroxidase | Brown, 2009 |
| *NDE1* | orf19.339 | *NDE1* | 2,5 | 2,7 |  | Putative NADH dehydrogenase | Brown, 2009 |
| *CAN2* | orf19.111 | *CAN1* | 2,45 |  |  | arginine permease | Martchenko, 2007 |
| *HSP12* | orf19.3160 | *HSP12* | 2,41 |  |  | Heat-shock protein; molecular chaperone | Martchenko, 2007 |
| *C2_08440W_A* | orf19.3637 | */* | 2,4 |  |  | Hypothetical protein | Martchenko, 2007 |
| *C4_03370C_A* | orf19.3364 | */* | 2,4 | 2,9 |  | ORF, Uncharacterized | Brown, 2009 |
| *GLK4* | orf19.6116 | *GLK1* | 2,4 |  |  | aldohexose specific glucokinase | Martchenko, 2007 |
| *ROD1* | orf19.1509 | *ROD1* | 2,4 | 3,8 |  | Drug tolerance, Rgt1-repressed | Brown, 2009 |
| *FCR1* | orf19.6817 | *CAT8* | 2,3 | 2,8 |  | Put. Zn-cluster transcription factor | Brown, 2009 |
| *GPH1* | orf19.7021 | *GPH1* | 2,3 |  |  | Glycogen phosphorylase | Martchenko, 2007 |
| *GPX3* | orf19.4436 | *GPX2* | 2,3 | 2,4 |  | Putative glutathione peroxidase | Brown, 2009 |
| *STB3* | orf19.203 | *STB3* | 2,3 | 1,4 |  | Predicted Sin3 binding protein | Brown, 2009 |
| *ALS2* | orf19.1097 | *SAG1* | 2,2 |  |  | Cell-wall protein | Martchenko, 2007 |
| *C1_01930W_A* | orf19.4530 | *YIL024C* | 2,2 | 2,9 |  | Protein of unknown function | Brown, 2009 |
| *C1_05540C_A* | orf19.411 | */* | 2,2 | 4,1 |  | Protein similar to GTPase regulators | Brown, 2009 |
| *CR_04680C_A* | orf19.1736 | */* | 2,2 | 2,7 |  | Protein of unknown function | Brown, 2009 |
| *MAK3* | orf19.4617 | *MAK3* | 2,2 | 6,2 |  | Predicted peptide alpha-N-acetyltransferase | Brown, 2009 |
| *C1_01840C_A* | orf19.4539 | *RDI1* | 2,19 |  |  | Putative rho GDP dissociation inhibitor | Martchenko, 2007 |
| *C4_05360C_A* | orf19.1794 | *PET54* | 2,19 |  |  | Hypothetical protein | Martchenko, 2007 |
| *C1_08770W_A* | orf19.4735 | *YGL159W* | 2,17 |  |  | Putative ornithine cyclodeaminase | Martchenko, 2007 |
| *PCK1* | orf19.7514 | *PCK1* | 2,16 |  |  | Phosphoenolpyruvate carboxykinase | Martchenko, 2007 |
| *SOU1* | orf19.2896 | *SPS19* | 2,11 |  |  | Peroxisomal dienoyl-CoA reductase, sorbitol utilization | Martchenko, 2007 |
| *C2_00770W_A* | orf19.2047 | *YOR289W* | 2,1 | 3,5 |  | Putative protein of unknown function | Brown, 2009 |
| *C7_00310C_A* | orf19.7091 | */* | 2,1 | 2,3 |  | Protein of unknown function | Brown, 2009 |
| *OPT9* | orf19.2584 | *OPT1* | 2,1 | 3,1 |  | Probable pseudogene | Brown, 2009 |
| *PTC8* | orf19.4698 | */* | 2,1 | 2,4 |  | Predicted type 2C protein phosphatase | Brown, 2009 |
| *QTH1* | orf19.6899 | */* | 2,1 | 2,3 |  | Putative oxidoreductase | Brown, 2009 |
| *XKS1* | orf19.1290 | *XKS1* | 2,1 | 2,8 |  | Putative xylulokinase | Brown, 2009 |
| *C3_01130C_A* | orf19.2515 | */* | 2,09 |  |  | Hypothetical protein | Martchenko, 2007 |
| *OYE32* | orf19.3131 | *OYE3* | 2,09 |  |  | NADPH dehydrogenase | Martchenko, 2007 |
| *SPS19* | orf19.3684 | *SPS19* | 2,08 |  |  | Peroxisomal 2,4-dienoyl-CoA reductase | Martchenko, 2007 |
| *PUT1* | orf19.4274 | *PUT1* | 2,05 |  |  | Proline oxidase | Martchenko, 2007 |
| *UGA1* | orf19.802 | *UGA1* | 2,05 |  |  | 4-aminobutyrate aminotransferase (nitrogen) | Martchenko, 2007 |
| *ARO10* | orf19.1847 | *ARO10* | 2,03 |  |  | Protein described as pyruvate decarboylase, LEU catabolism | Martchenko, 2007 |
| *ALS4* | orf19.2121 | */* | 2,02 |  |  | Cell wall protein | Martchenko, 2007 |
| *CTN1* | orf19.4551 | *YAT1* | 2,01 |  |  | Mitochondrial carnitine acetyltransferase | Martchenko, 2007 |
| *GDB1* | orf19.744 | *GDB1* | 2,01 |  |  | Glycogen debranching enzyme | Martchenko, 2007 |
| *ARG4* | orf19.6689 | *ARG4* | 2 | 3,9 |  | Argininosuccinate lyase | Brown, 2009 |
| *ARG5* | orf19.4788 | *ARG5,6* | 2 | 5 |  | Arginine biosynthetic enzyme | Brown, 2009 |
| *C1_11270W_A* | orf19.675 | */* | 2 | 2,1 |  | Putative fungal-specific transmembrane protein | Brown, 2009 |
| *C7_01690W_A* | orf19.6555 | *HOT13* | 2 | 2 |  | Ortholog(s) have zinc ion binding activity | Brown, 2009 |
| *EBP7* | orf19.5816 | *OYE3* | 2 | 1,9 |  | Stress-induced via Cap1p | Brown, 2009 |
| *ORF19.3070.1* | orf19.3070.1 | */* | 2 |  |  | Hydratase-dehydrogenase-epimerase | Martchenko, 2007 |
| *ACS1* | orf19.1743 | *ACS1* | 1,93 |  |  | Acetyl-coenzyme A synthetase | Martchenko, 2007 |
| *GRE3* | orf19.4317 | *GRE3* | 1,93 |  |  | Aldose reductase | Martchenko, 2007 |
| *WH11* | orf19.3548.1 | *HSP12* | 1,92 |  |  | Heat-shock protein | Martchenko, 2007 |
| *HGT17* | orf19.4682 | *HXT17* | 1,91 |  |  | Quinate permease | Martchenko, 2007 |
| *HRQ2* | orf19.5037 | */* | 1,91 |  |  | Conserved hypothetical protein | Martchenko, 2007 |
| *C6_02560W_A* | orf19.5525 | *YMR315W* | 1,9 |  |  | Conserved hypothetical protein | Martchenko, 2007 |
| *DOG1* | orf19.3392 | *DOG2* | 1,9 | 2,6 |  | Put. 2-deoxygluc-6-phophatase | Brown, 2009 |
| *ORF19.682* | orf19.682 | */* | 1,9 |  |  | Hypothetical protein | Martchenko, 2007 |
| *YIM1* | orf19.847 | *YIM1* | 1,9 | 1,8 |  | Similar to mitochondrial protease | Brown, 2009 |
| *C2_07630C_A* | orf19.1862 | *RTC3* | 1,83 |  |  | Conserved hypothetical protein | Martchenko, 2007 |
| *GNP1* | orf19.7566 | *AGP1* | 1,82 |  |  | High-affinity glutamine permease | Martchenko, 2007 |
| *C3_03410C_A* | orf19.338 | *YMR196W* | 1,78 |  |  | Conserved hypothetical protein | Martchenko, 2007 |
| *FAA21* | orf19.272 | *FAA2* | 1,74 |  |  | Long-chain fatty acid Coa ligase | Martchenko, 2007 |
| *TFS1* | orf19.1974 | *TFS1* | 1,73 |  |  | Carboxypeptidase Y inhibitor | Martchenko, 2007 |
| *POT1* | orf19.7520 | *POT1* | 1,71 |  |  | Peroxysomal 3-ketoacyl-CoA thiolase A | Martchenko, 2007 |
| *MDR1* | orf19.5604 | *FLR1* | 1,7 |  |  | Benomyl/methotrexate resistance protein | Martchenko, 2007 |
| *CR_00090C_A* | orf19.7531 | *YMR090W* | 1,69 |  |  | Conserved hypothetical protein | Martchenko, 2007 |
| *C1_00200C_A* | orf19.6083 | */* | 1,68 |  |  | hypothetical protein | Martchenko, 2007 |
| *C1_11670W_A* | orf19.1152 | */* | 1,67 |  |  | Hyptothetical protein | Martchenko, 2007 |
| *GSY1* | orf19.3278 | *GSY1* | 1,67 |  |  | Glycogen (starch) synthase | Martchenko, 2007 |
| *CSH1* | orf19.4477 | *YPL088W* | 1,66 |  |  | Aryl-alcohol dehydrogenase | Martchenko, 2007 |
| *XKS1* | orf19.1788 | */* | 1,66 |  |  | Xylulokinase | Martchenko, 2007 |
| *CYB2* | orf19.5000 | *CYB2* | 1,64 |  |  | Cytochrome b2 precursor | Martchenko, 2007 |
| *RBR2* | orf19.532 | *TIP1* | 1,64 |  |  | Cell-wall protein | Martchenko, 2007 |
| *PDK2* | orf19.7281 | *PKP1* | 1,63 |  |  | Pyruvate dehydrogenase kinase | Martchenko, 2007 |
| *BZD99* | orf19.4287 | *XYL2* | 1,61 |  |  | Alcohol dehydrogenase (glucose catabolism to butanediol) | Martchenko, 2007 |
| *MRF1* | orf19.1149 | *ETR1* | 1,61 |  |  | Mitochondrial 2-enoyl thioester reductase, respiration | Martchenko, 2007 |
| *GLG21* | orf19.3325 | *GLG2* | 1,6 |  |  | Self-glucosylating initiator of glycogen synthesis | Martchenko, 2007 |
| *C6_00210W_A* | orf19.1180 | *YER152C* | 1,59 |  |  | Conserved hypothetical protein | Martchenko, 2007 |
| *FOX2* | orf19.1809 | */* | 1,59 |  |  | Peroxisomal hydratase-dehydrogensase-epimerase | Martchenko, 2007 |
| *UGA21* | orf19.345 | *UGA2* | 1,59 |  |  | Succinate semialdehyde dehydrogenase | Martchenko, 2007 |
| *ECM4* | orf19.2613 | *ECM4* | 1,57 |  |  | Involved in cell-wall biogenesis and architecture | Martchenko, 2007 |
| *DCI1* | orf19.6443 | *ECI1* | 1,56 |  |  | Enoyl-CoA isomerase | Martchenko, 2007 |
| *XYL2* | orf19.7676 | *SOR2* | 1,56 |  |  | Sorbitol dehydrogenase | Martchenko, 2007 |
| *C4_02620C_A* | orf19.2737 | *YDR109C* | 1,55 |  |  | Kinase | Martchenko, 2007 |
| *GSF2* | orf19.6082 | *GSF2* | 1,55 |  |  | ER localized promote secretion of GAL2 | Martchenko, 2007 |
| *PEX5* | orf19.5640 | *PEX5* | 1,55 |  |  | Peroxisomal protein receptor | Martchenko, 2007 |
| *EHD3* | orf19.3029 | *EHD3* | 1,54 |  |  | Enpyl-CoA hydratase | Martchenko, 2007 |
| *CR_05750W_A* | orf19.6637 | */* | 1,53 |  |  | Predicted glycosilase | Martchenko, 2007 |
| *ECM38* | orf19.1325 | */* | 1,52 |  |  | Gamma-glutamyltransferase, cell wall organization | Martchenko, 2007 |
| *UGA11* | orf19.854 | *UGA1* | 1,52 |  |  | 4-aminobutyrate aminotransferase (GABA transaminase) | Martchenko, 2007 |
| *POX1-3* | orf19.1652 | */* | 1,51 |  |  | Fatty-acyl coenzyme A oxidase | Martchenko, 2007 |
| *NAG1* | orf19.2156 | */* |  |  | 5,699282607 | Glucosamine-6-phosphate deaminase | Gunasekera, 2010 |
| *DAC1* | orf19.2157 | */* |  |  | 5,536242867 | N-acetylglucosamine-6-phosphate (GlcNAcP) deacetylase | Gunasekera, 2010 |
| *HXK1* | orf19.2154 | *HXK1* |  |  | 4,147854725 | N-acetylglucosamine (GlcNAc) kinase | Gunasekera, 2010 |
| *NGT1* | orf19.5392 | */* |  |  | 3,992962683 | N-acetylglucosamine (GlcNAc)-specific transporter | Gunasekera, 2010 |
| *GIG1* | orf19.1066 | *YPL067C* |  |  | 3,564433304 | Protein induced by N-acetylglucosamine (GlcNAc) | Gunasekera, 2010 |
| *ECE1* | orf19.3374 | */* |  |  | 3,442074601 | Candidalysin | Gunasekera, 2010 |
| *PGA34* | orf19.2833 | */* |  |  | 3,23679831 | Putative GPI-anchored protein | Gunasekera, 2010 |
| *SAP6* | orf19.5542 | *BAR1* |  |  | 2,942374866 | Biofilm-specific aspartyl protease | Gunasekera, 2010 |
| *HWP1* | orf19.1321 | */* |  |  | 2,416430129 | Hyphal cell wall protein | Gunasekera, 2010 |
| *ECM331* | orf19.4255 | *ECM33* |  |  | 2,414427653 | GPI-anchored protein | Gunasekera, 2010 |
| *RBT4* | orf19.6202 | *PRY3* |  |  | 2,273053313 | Pry family protein | Gunasekera, 2010 |
| *C1_05920W_A* | orf19.2457 | */* |  |  | 2,229461027 | Protein of unknown function | Gunasekera, 2010 |
| *PEX25* | orf19.5575 | *PEX25* |  |  | 2,196759392 | Putative peripheral peroxisomal membrane peroxin | Gunasekera, 2010 |
| *SAP5* | orf19.5585 | *YPS3* |  |  | 2,148159351 | Biofilm-specific aspartyl protease | Gunasekera, 2010 |
| *C5_03710C_A* | orf19.1124 | */* |  |  | 2,098062888 | Protein of unknown function | Gunasekera, 2010 |
| *HGC1* | orf19.6028 | *CLN2* |  |  | 2,052328433 | Hypha-specific G1 cyclin-related protein involved in regulation of morphogenesis | Gunasekera, 2010 |
| *FAV2* | orf19.1120 | */* |  |  | 2,03070729 | Adhesin-like protein | Gunasekera, 2010 |
| *IHD1* | orf19.5760 | *YFL067W* |  |  | 1,896519023 | GPI-anchored protein | Gunasekera, 2010 |
| *DDD1* | orf19.2036 | */* |  |  | 1,81877949 | Predicted dihydrodiol dehydrogenase | Gunasekera, 2010 |
| *CFL11* | orf19.701 | *FRE3* |  |  | 1,797934917 | Superoxide-generating NADPH oxidase | Gunasekera, 2010 |
| *ALS10* | orf19.2355 | */* |  |  | 1,792695154 | ALS family cell-surface glycoprotein | Gunasekera, 2010 |
| *ALS3* | orf19.1816 | *SAG1* |  |  | 1,75708255 | Cell wall adhesin | Gunasekera, 2010 |
| *GIT2* | orf19.1978 | *GIT1* |  |  | 1,723746257 | Putative glycerophosphoinositol permease | Gunasekera, 2010 |
| *GAL102* | orf19.3674 | *GAL10* |  |  | 1,714139892 | UDP-glucose 4,6-dehydratase | Gunasekera, 2010 |
| *HYR1* | orf19.4975 | */* |  |  | 1,665839679 | GPI-anchored hyphal cell wall protein | Gunasekera, 2010 |
| *RFX2* | orf19.4590 | *RFX1* |  |  | 1,573054621 | Transcriptional repressor | Gunasekera, 2010 |
| *C6_01360W_A* | orf19.3448 | */* |  |  | 1,565580448 | Protein of unknown function | Gunasekera, 2010 |
| *RBT1* | orf19.1327 | */* |  |  | 1,562233216 | Cell wall protein with similarity to Hwp1 | Gunasekera, 2010 |
| *RBT1* | orf19.3384 | */* |  |  | 1,503588143 | Putative cell wall protein with C-terminal similarity to Hwp1 | Gunasekera, 2010 |
| *ARF3* | orf19.1702 | *ARF3* |  |  | 1,482241442 | Similar to but not orthologous to *S. cerevisiae* Arf3 | Gunasekera, 2010 |
| *PHR1* | orf19.3829 | *GAS1* |  |  | 1,456980867 | Cell surface glycosidase | Gunasekera, 2010 |
| *DEF1* | orf19.7561 | */* |  |  | 1,450666597 | RNA polymerase regulator II | Gunasekera, 2010 |
| *IAH1* | orf19.7667 | *IAH1* |  |  | 1,420739666 | Protein similar to *S. cerevisiae* Iah1p | Gunasekera, 2010 |
| *FMA1* | orf19.5193 | */* |  |  | 1,402505558 | Protein similar to oxidoreductases | Gunasekera, 2010 |
| *IDH2* | orf19.6021 | */* |  |  | 1,394038665 | Protein of unknown function | Gunasekera, 2010 |
| *UME6* | orf19.1822 | *UME6* |  |  | 1,377614355 | Zn(II)2Cys6 transcription factor | Gunasekera, 2010 |
| *TPO4* | orf19.473 | *TPO4* |  |  | 1,37354051 | Putative sperimidine transporter | Gunasekera, 2010 |
| *GCN4* | orf19.1358 | *GCN4* |  |  | 1,346876538 | bZIP transcription factor | Gunasekera, 2010 |
| *SNO1* | orf19.2948 | *SNO1* |  |  | 1,342190427 | Protein with a predicted role in pyridoxine metabolism | Gunasekera, 2010 |
| *CR_09940W_A* | orf19.7568 | *SPS4* |  |  | 1,302503325 | Ortholog of *S. cerevisiae* *SPS4* | Gunasekera, 2010 |
| *orf19.6363* | orf19.6363 | */* |  |  | 1,301214357 | Predicted ORF | Gunasekera, 2010 |

## Supplementary table 1

The table shows the genes upregulated in *C. albicans* in the presence of galactose, glucose and/or N-acetylglucosamine. The first column indicates the gene name. The second column indicates the ORF ID found in the *Candida* genome database (http://www.candidagenome.org/). The third column shows the ortholog in *S. cerevisiae*. The fourth, fifth and sixth column shows the gene expression in response to galactose, glucose and N-acetylglucosamine respectively relative to different conditions. The data from Brown, et al., 2009 are represented on the left side of the fourth column and in the fifth column and show the increased expression upon galactose and glucose addition respectively relative to cells grown in glycerol. The data from Martchenko, et al., 2007 are shown in the fourth column on the right side and represent the increased expression upon galactose addition to overnight grown cells. The data from Gunasekera, et al., 2010 are shown in the sixth column and represent the increased expression upon 5 mM GlcNAc addition relative to cells grown in the presence of 2% glucose. The seventh column shows the (presumed) function of the gene products. The eighth column shows the reference of which the data were adapted from. The data are listed showing induced expression upon galactose addition in descending order.
